# Clinical utility of microRNA-378 as early diagnostic biomarker of human cancers: a meta-analysis of diagnostic test

**DOI:** 10.18632/oncotarget.10707

**Published:** 2016-07-19

**Authors:** Zhan-Zhan Li, Liang-Fang Shen, Yan-Yan Li, Peng Chen, Li-Zhang Chen

**Affiliations:** ^1^ Department of Oncology, Xiangya Hospital, Central South University, Changsha, Hunan Province 410008, China; ^2^ Department of Nursing, Xiangya Hospital, Central South University, Changsha, Hunan Province 410008, China; ^3^ Department of Orthopedics, Xiangya Hospital, Central South University, Changsha, Hunan Province 410008, China; ^4^ Department of Epidemiology and Health Statistics, School of Public Health, Central South University, Changsha, Hunan Province 410078, China

**Keywords:** cancer, microRNA-378, early diagnosis, meta-analysis, tumor marker

## Abstract

A meta-analysis was performed to evaluate the diagnostic value of miR-378 for detecting human cancers. Systematic electronic searches were conducted in PubMed, Web of Science, Embase, China National Knowledge Infrastructure, and Wanfang from the inception to January 15, 2016. We used the bivariate mixed effects models to estimate the combined sensitivity, specificity, PLRs (positive likelihood ratios), NLR (negative likelihood ratios), DORs (diagnostic odds ratios) and their 95% CI (confidence intervals) for assessing the diagnostic performance of miR-378 for cancers. Twelve studies were included in the meta-analysis, with a total number of 1172 cancer patients and 809 health controls. The overall estimated sensitivity and specificity were 0.75 and 0.74. The pooled PLR was 2.91, NLR was 0.34, DOR was 8.50, and AUC (Area Under the Curve) was 0.81. The subgroup analyses suggested that AUC for plasma-based is higher than serum-based. The overall diagnostic values of miR-378 in the present meta-analyses are moderate accurate for human cancers; The source of specimen has an effect on the diagnostic accuracy. The diagnostic value of serum-based was higher than that of plasma-based.

## INTRODUCTION

Cancers have become an important public issue on a world scale. According to the World Cancer Research Fund report in 2014, the newly added cancer patients had 1400 million worldwide [1]. Previous studies had suggested that many reasons could lead to cancer occurrence such as unhealthy lifestyles, environment exposing, and special eating habitats [2–4]. With the rising mortality caused by cancers, the prognosis and survival situation of the cancer patients are not optimistic. It was reported that the five-year survival rate for cancer patients was about 50% [5]. But the 5-year survival rate for cancer patients after diagnosis and treatments strongly depends on the types of cancer. Patients with prostate cancer have a more than 80% chance of survival past 5 years while the 5-year survival rate for pancreatic patients is less than 6% [6, 7]. Some other cancers also have low 5-year survival rates such as 5–9% for hepatocellular carcinoma patients [8], 13% for lung cancer patients [9], and 9.8% for patients with central nervous systems tumors [10].

Successful treatment of cancer patients, which largely depends on early detection, is important to improve survival rates and life qualities of patients with cancers. At present, the most common method to diagnosis cancers is histopathological examination. However, the invasive procedure restricts its application [11]. The markers examination of circulating blood in detecting cancers (serum/plasma) received widespread attention because of its less trauma and acceptability for cancer patients. The α-fetoprotein (AFP), carcinoembryonic antigen (CEA), and carbohydrate antigen (CA) are the most commonly used serum markers for early detection of cancers. These biomarkers do not meet the requirements of clinical practice and population screening because of their low sensitivity or specificity and expenses, especially for a wide screening for national level population [12]. Therefore, a simple, quick and sufficient marker for detecting cancers are urgently needed. MicroRNAs or miRNAs, non-coding RNAS with 17–25 nucleotides, are small endogenous RNAs. The miRNAs are found in a variety of biological cells and evolutionarily conserved [13]. It is suggested that the high or low expression miRNAs are detected in different kinds of human cancers, the expression profiles and levels exhibit an apparent tissue specificity and time phases, and miRNAs can resist enzyme degradation. The most importance is that miRNAs also can stably exist in the circulating blood because of their particular structure [14], which are ideal biomarkers of detecting cancers. The miRNA-378 is an important tumor-related gene regulatory site. Previous study found that the miRNA-378 expression levels could identify cancer patients and health individuals [15]. However, the diagnostic values of miRNA-378 remain inconsistent in different studies, which could be caused by the limitation of sample size, study group and cancers types [16, 17]. We conducted a meta-analysis to evaluate the diagnostic values of miRNA-378 for detecting human cancers.

## RESULTS

### Study selection

**Figure 1:**
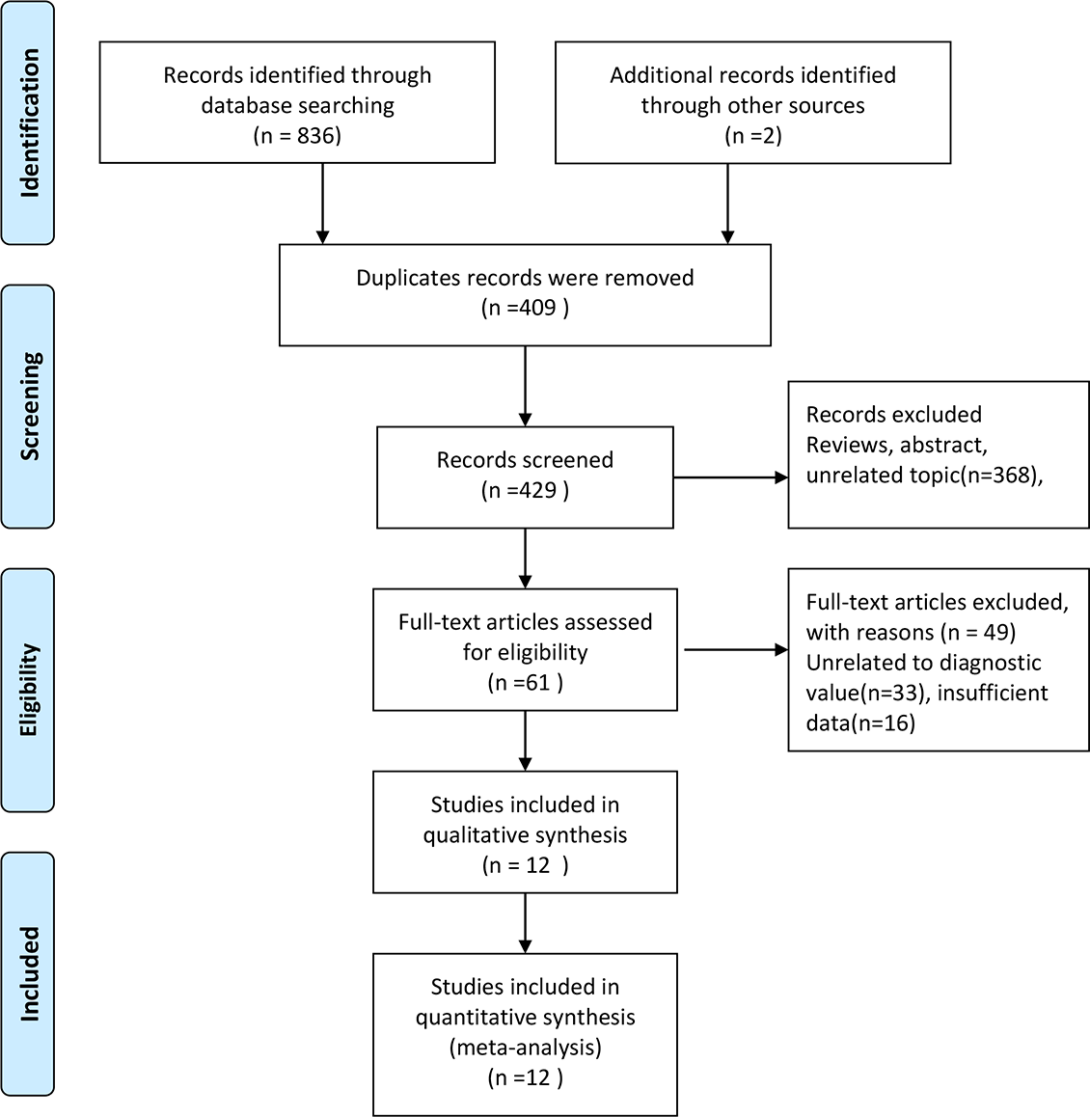
Flow diagram of studies selection process

Figure 1 shows the process of literature search and screening. The initial search returned 836 records and 2 records were obtained through manual retrieval. 429 records were left for further screening after removing the duplicated records. After reviewing abstracts and titles, we excluded 368 records and 61 articles were thought to be potentially eligible for inclusion. We excluded 49 records because the following reasons: 33 records with unrelated to diagnostic values or other miRNAs, and 16 insufficient data. Finally, 12 studies were included in the meta-analysis [16–27]. One of them included four groups of data [25].

### Study characteristics

The Table 1 summarized the main characteristics of the included studies. These studies were published from 2013 to 2015. The sample sizes ranged from 42 to 295, with a total number of 1172 cancer patients and 809 health controls. In the present studies, the types of cancers included renal cell cancer (RCC), gastric cancer (GC), nasopharyngeal carcinoma (NC), colorectal carcinoma (CC). breast cancer (BC), pancreatic cancer(PC). Among the 12 studies, 7 studies were conducted in Asian population, and 5 studies were from Caucasian population. Six studies samples were from serum, six were from plasma, and two were from tissues. All miRNAs were detected through quantificational real-time polymerase chain reaction (qRT-PCR).

**Table 1:** Characteristics of the included studies in the meta-analysis of miR-378 for cancer detection

Author	Year	Country	Ethnicity	Case	Control	Type	Sample	Methods of detection	TP	FP	FN	TN
Redova	2012	Germany	Caucasian	90	35	RCC	Serum	qRT-PCR	63	14	27	21
Peng	2015	China	Asian	32	32	HCC	Tissue	qRT-PCR	29	8	2	23
Liu	2012	China	Asian	61	61	GC	Serum	qRT-PCR	53	18	8	43
Zanutto	2014	Italy	Caucasian	29	29	CC	Plasma	qRT-PCR	23	8	6	21
Liu	2013	China	Asian	217	73	NC	Plasma	qRT-PCR	146	29	71	44
Yin	2014	China	Asian	101	40	BC	Tissue	qRT-PCR	69	4	32	36
Fedorko	2015	Czech	Caucasian	195	100	RCC	Serum	qRT-PCR	159	17	36	83
Li	2013	America	Caucasian	41	19	PC	Serum	qRT-PCR	31	4	10	15
Hauser	2012	Germany	Caucasian	25	25	RCC	Serum	qRT-PCR	18	16	7	19
Wang	2015	China	Asian	107	107	RCC	Serum	qRT-PCR	79	28	26	81
Wang^a^	2015	China	Asian	28	28	RCC	Serum	qRT-PCR	20	8	7	21
Wang^b^	2015	China	Asian	79	79	RCC	Serum	qRT-PCR	63	16	21	58
Wang^c^	2015	China	Asian	76	107	RCC	Serum	qRT-PCR	54	22	21	86
Li	2015	China	Asian	22	20	RCC	Plasma	qRT-PCR	15	2	7	18
Li	2013	China	Asian	69	54	GC	Plasma	qRT-PCR	45	13	24	32

RCC, renal cell carcinoma; HCC, hepatocellular carcinoma; CC, colorectal carcinoma; BC, breast cancer; PC, pancreatic cancer; NC, nasopharyngeal carcinoma; GC, gastric cancer; qRT-PCR, quantificational real-time polymerase chain reaction.

### Assessment of quality

The overview of the quality of included were presented in Supplementary Figure S1 and Supplementary Figure S2. Four studies given unclear description for patient selection. The index test was judged as unclear in six studies that did not confirm whether the results were interpreted without knowledge of the results of the reference standard as well as reference standard. Five studies without avoided case-control design, inappropriate exclusions or interpreted results with knowledge of the results of the references standard were considered as having a high risk bias. For applicability concerns, each of three domain keys has one study that given unclear description.

### Pooled diagnostic values

**Figure 2:**
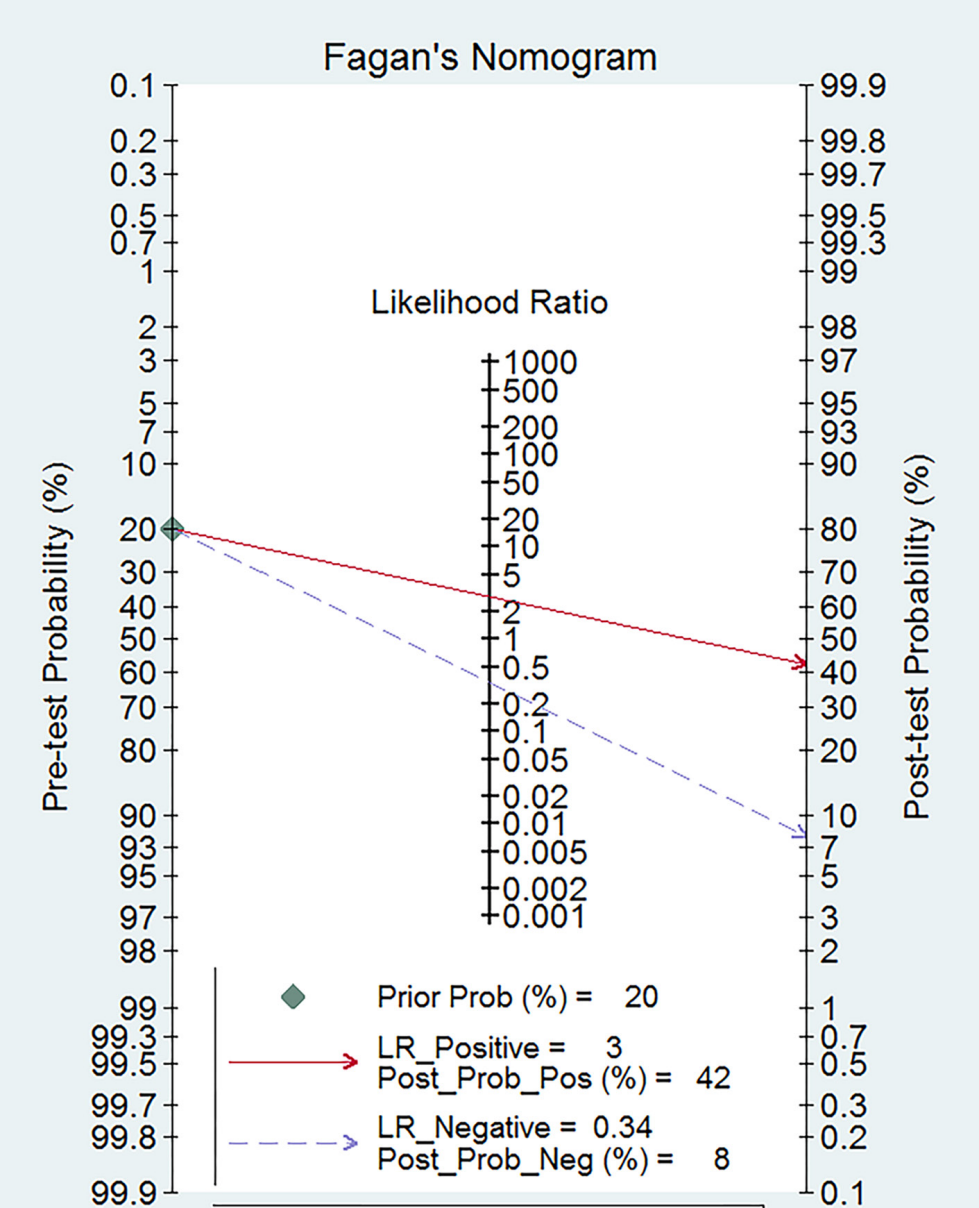
Fagan diagram evaluating the overall diagnostic value of miR-378 for cancer

**Figure 3:**
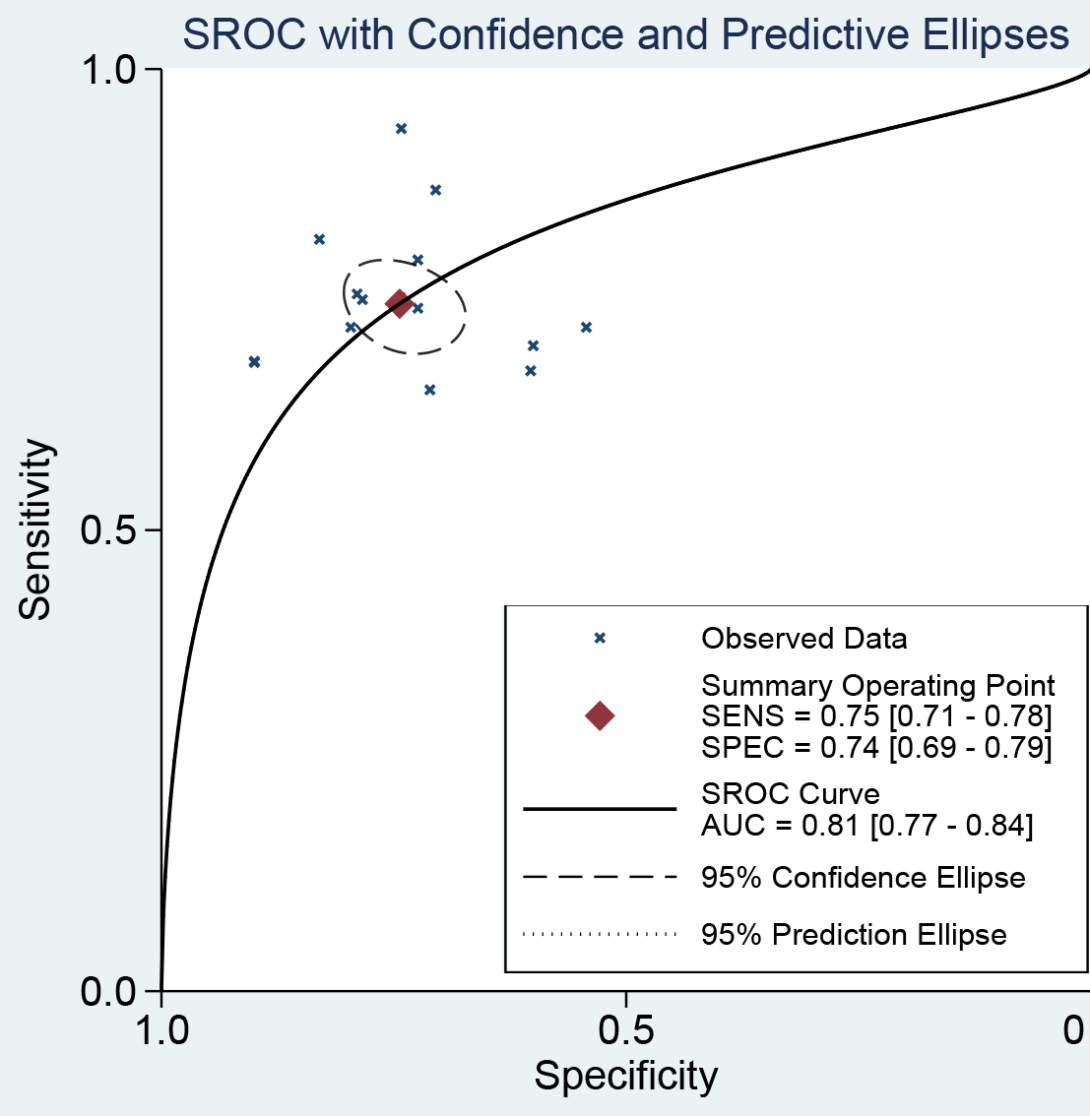
The SROC curve of miR-378 test for the diagnosis of various cancers

The Spearman coefficient was −0.082 and *P* = 0.770, which means no threshold effect. The *I*^2^ values for sensitivity and specificity were more than 50%, and random effect models were used. The estimated diagnostic values of miR-378 for detecting cancers are shown in Table 2. The overall estimated sensitivity and specificity were 0.75 (95% CI: 0.71–0.78) and 0.74 (95% CI: 0.69–0.79). The pooled PLR was 2.91 (95% CI:2.38–3.55), NLR was 0.34 (95% CI:0.29–0.41), and DOR was 8.50 (95% CI: 6.01–12.01). Fangan plot was shown in Figure 2. The overall SROC curve was shown in Figure 2, Figure 3 and AUC was 0.81 (95% CI: 0.77–0.84). The diagnostic accuracy of miR-378 for cancers was relatively high.

**Table 2:** Summary estimated of diagnostic performance of miR-378 for cancer detection

Category	Cases/controls	SEN (95% CI)	SPE (95% CI)	PLR (95% CI)	NLR (95% CI)	DOR (95% CI)	AUC (95% CI)
**Overall**	1172/809	0.75 [0.71–0.78]	0.74 [0.69–0.79]	2.91 [2.38–3.55]	0.34 [0.29–0.41]	8.50 [6.01–12.01]	0.81 [0.77–0.84]
**Ethnicity**							
Asian	792/601	0.74 [0.69–0.79]	0.76 [0.70–0.80]	3.02 [2.44–3.75]	0.35 [0.28–0.42]	8.77 [6.01–12.80]	0.81 [0.78–.84]
Caucasian	380/208	0.76 [0.69–0.81]	0.71 [0.59–0.80]	2.60 [1.72–3.94]	0.34 [0.24–0.48]	7.62 [3.63–16.03]	0.80 [0.76–0.83]
**Cancer Type**							
Renal cell carcinoma	622/501	0.75 [0.71–0.78]	0.74 [0.69–0.79]	2.91 [2.38–3.55]	0.34 [0.29–0.41]	8.50 [6.01–12.01]	0.81 [0.77–0.86]
Other types	550/308	0.76 [0.68–0.83]	0.73 [0.66–0.8]	2.87 [2.13–3.85]	0.32 [0.23–0.46]	8.89 [4.95–15.92]	0.81 [0.77–0.84]
**Sample types**							
Serum–based	702/561	0.75 [0.71–0.79]	0.74 [0.68–0.80]	2.91 [2.22–3.81]	0.34 [0.27–0.42]	8.65 [5.45–13.73]	0.80 [0.77–0.84]
Plasma–based	337/176	0.68 [0.62–0.74]	0.72 [0.59–0.81]	2.38 [1.59–3.55]	0.44 [0.34–0.58]	5.31 [2.84–10.13]	0.70 [0.66–0.74]

### Subgroup analyses

**Figure 4:**
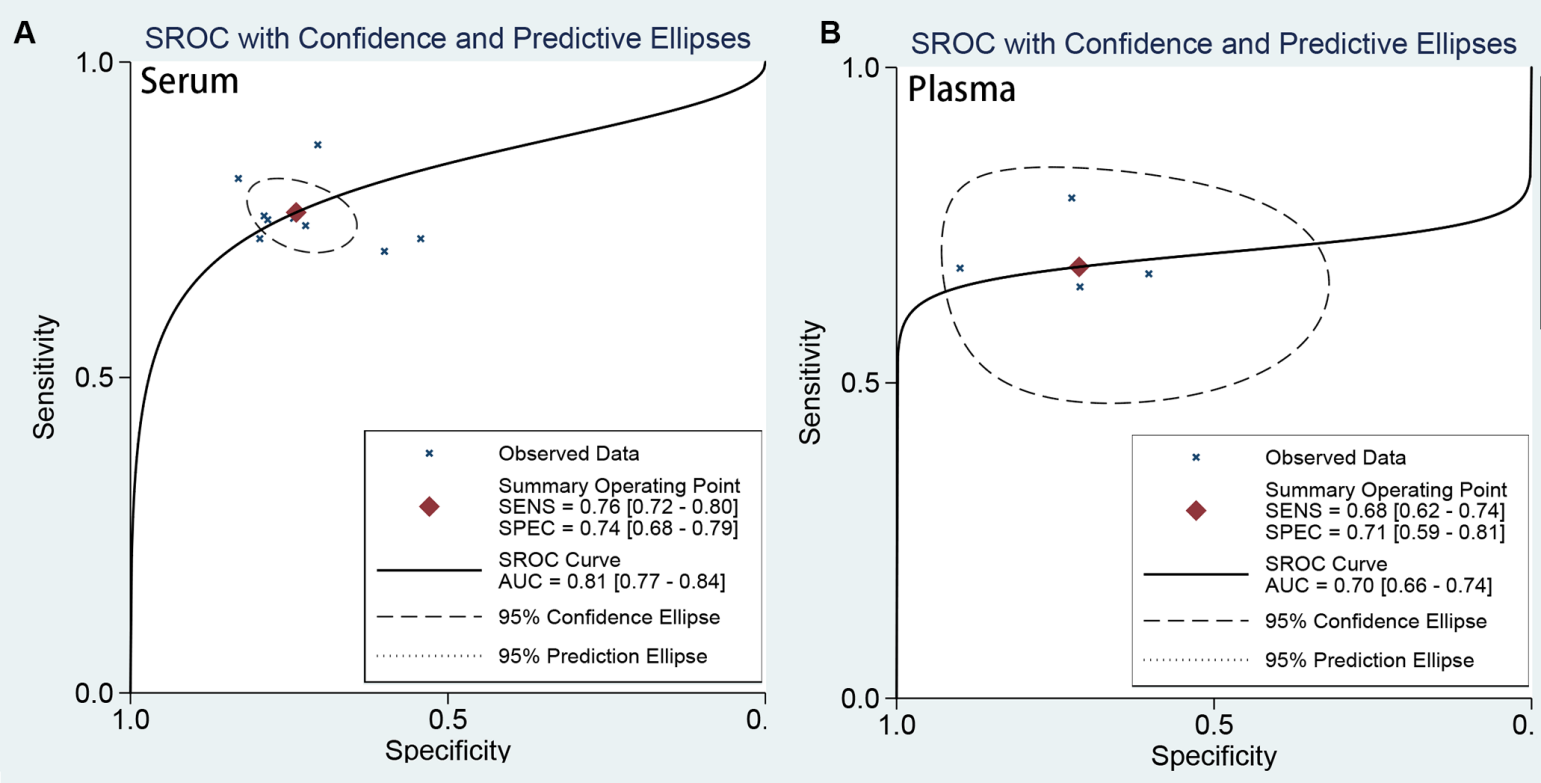
The SROC curve of miR-378 test for the diagnosis of various cancers (A) SROC curve of serum-based; (B) SROC curve of plasma-based)

Subgroup analyses were conducted in the ethnicity (Asian vs Caucasian), cancer type (renal cell carcinoma vs other types), sample types (serum-based, plasma-based and) setting. The subgroup results of all estimates were presented in Table 2 (sensitivity, specificity, PLR, NLR, and DOR). There are no obvious differences between combined results in the ethnicity and cancer type. The pooled sensitivity and specificity and AUC were similar (Table 2). The results suggested that ethnicity may be not an influence factor on heterogeneity. However, the sample types indicated significant difference of estimated results. The AUC for plasma-based (0.80, 0.77–0.84) is significantly different from serum-based (0.70, 0.66–0.74) in Figure 4. The diagnostic accuracy of serum-based is higher than that of plasma-based. It suggests that the diagnostic cutoff values of miR-378 for detecting cancers is correlate to source of samples. The rest results were similar. The pooled sensitivity, specificity, PLR, and NLR for serum-based were: 0.75 (95% CI: 0.71–0.79), 0.74 (95% CI: 0.68–0.80), 2.91 (95% CI: 2.22–3.81), 0.34 (95% CI: 0.27–0.42); For plasma-based: 0.68 (95% CI: 0.62–0.74), 0.72 (95% CI: 0.59–0.81), 2.38 (95% CI: 1.59–3.55), and 0.44 (95% CI: 0.34–0.58).

### Sensitivity analyses and publication bias

**Figure 5:**
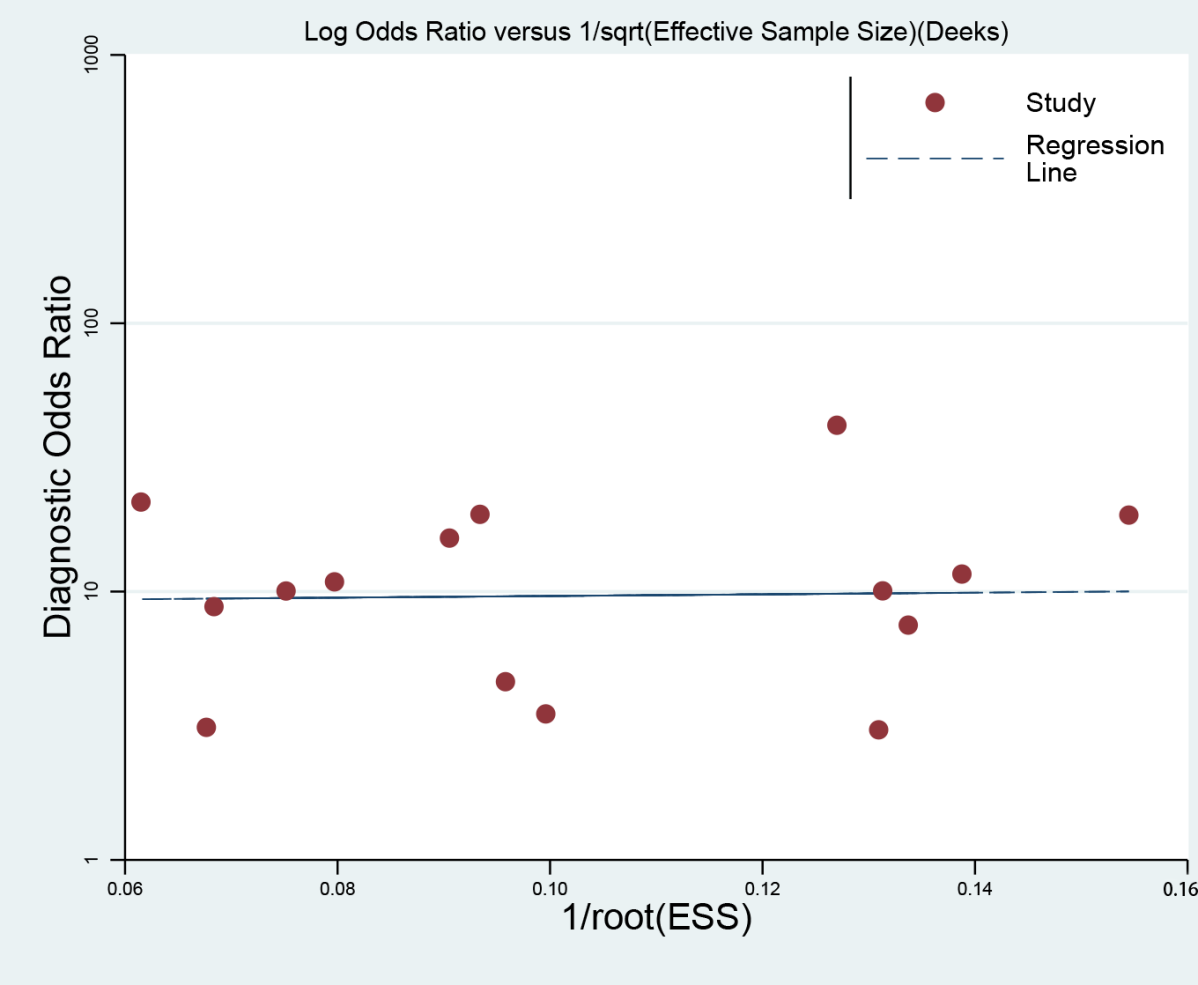
Deek's funnel plot to evaluate the publication bias

We conducted sensitivity analyses through sequentially excluding individual studies, and the summary sensitivity and specificity, PLR, NLR and ACU were altered (data were not given), indicating that the present pooled estimated were stable. We used Deek's plot to evaluate the publication bias. The bias test shown no existence of publication bias (*t* = 0.09, *P* = 0.929) as indicated in Figure 5.

## DISCUSSION

In the present meta-analysis, twelve studies were included, and the pooled results including all studies showed miRNA-378 gave an AUC of 0.81 (95% CI:0.77–0.84) with a sensitivity value of 75% and specificity of 74% in identifying the patients with cancers from health individuals. The miRNA-378 showed a moderate accuracy in detecting cancer patients. We also found the source of specimen had an effect on the diagnostic value of miRNa-378. The specimen of serum-based is significantly higher than plasma-based specimen (AUC: 0.80, 0.77–0.84 vs 0.70, 0.66–0.74). The higher ACU value means better diagnostic ability of balance between sensitivity and specificity, especially for renal cell carcinoma.

The pooled diagnostic value of miR-378 is higher than traditional clinical markers such as CEA and CA19-9 [28], indicating the relatively high accuracy of miRNA-378 as an early diagnosing biomarker of cancers. Our results are almost equal to previous several miRNAs. Shen et al. conducted a meta-analysis of miRNA-21 in detecting human cancers. This study yielded a AUC of 0.88 with a sensitivity of 78% and a specificity of 83% for all cancers, and this study did not give the diagnostic value of RCC [29]. Another study conducted by Tang used different methods to evaluate the diagnostic value of all miRNAs for RCC. They used the hazard ratio to quantify the criteria of miRNAs instead of the sensitivity and specificity. The five miRNAs provided a reliable tool for RCC patients, especially for clear cell RCC [30]. Our results found that miRNA-378 had a AUC of 0.81 as well as other cancer types of the present results. The diagnostics value was similar to the oral squamous cell carcinoma (AUC of 0.832). This study did not focus on single miRNA but all miRNAs and gave an integrated miRNA expression profiling analysis [31]. The highest AUC in all included studies is 0.86 with 87.5% sensitivity and 70.73% specificity in diagnosing the GC patients [20], and the lowest AUC is 0.66 (95% CI: 0.57–0.77) for GC [27]. There are only two studies reporting the diagnostic value of GC, and further studies are needed.

miRNA-378 could promote the expression of proto-oncogenes through targeted localization and inhibiting the BTG (B-cell translocation gene) prohibiting. In addition, miR-378 could be the downstream targeted site of the c-Myc oncoprotein, which was involved in stable transfection of miR-378 resulted in cell survival, tumor growth and angiogenesis [32]. These possible mechanisms make miRNA-378 become a potential biomarker of detecting cancers. In the present meta-analysis, eight studies of the levels of miRNA-378 in the serum of RCC patients are included in the meta-analysis. Redoval found that the level of miRNA-378 increased in serum of RCC patients compared to healthy controls. miRNA-378 also was reported to be increased in GC and CRC patients [17, 20]. Not in parallel with these above results, Hause did not report the difference between RCC patients and controls [18, 24]. Another study by Wang even found that the miRNA-378 levels were significantly decreased in the serum of RCC patients [25], and decreased levels were also found in patients with NPC (nasopharyngeal carcinoma) [16]. Many factors could be attributed to the results differences from three studies such as population selection, sample size and disease types. Some authors speculated that cancer cells could intentionally release or capture miRNAs, and lead to elevated or decreased miRNAs levels in tumor tissues. The biological effects depended on cell-specific collection in target genes. It is reported that miRNA-378 could inhibit human GC MDC-803 cells by target MAPK1 *in vitro*, and promote BMP2-inducec osteogenic differentiation of mesenchymal progenitor cells [33]. Therefore, the single miRNA may have some limitations in detecting cancers, and the combination may provide more accurate diagnostic values.

The major strength of our study was that we strictly followed the PRISMA guidelines to conducted the meta-analysis, and evaluate the quality of include studies using the scale recommended by Cochrane Collaboration. There are still several limitations. First, in spite of the fact the present study yielded a moderate diagnostic value, we still recommend the combined miRNAs biomarkers to detect cancers. According to the criteria of high accuracy (PLR > 10, NLR < 0.1), the results of miR-378 are not high enough as expected. For clinical purpose, it is really necessary to make decisions combined with miRNAs. because it is reported that the combination of miRNA-371 with other miRNAs generate a more accurate result [18]. Second, there are seven types of cancer in the meta-analysis, and some cancer types are few, which makes our results more appropriate for detecting RCC. Further validation in large cohorts will be necessary. Third, the study subject of all include studies are Asian and Caucasian populations, and no studies with African population are included. The gene and mRNA could adjust the expression of protein. The mRNA could be an information carrier. The transcriptional regulation is the main way of gene expression, and post-transcriptional regulation also play an important role in the progression of gene expression. It is possible that different gene types could increase or decrease the expression of certain protein, which will lead to the occurrence of disease. Finally, our results showed that the diagnostic accuracy of serum-based specimen will be better than plasma-based in the overall cancers. Considering the different function of miRNAs, the value could be different in a specific caner type.

In conclusions, the overall diagnostic values of miR-378 in the present meta-analyses are moderate accurate for human cancers, especially for RCC; The source of specimen has an effect on the diagnostic accuracy. The diagnostic value of serum-based was higher than that of plasma-based. The future study should focus on the mechanism and combined effect of miRNA-378 and others miRNAs.

## MATERIALS AND METHODS

The present meta-analysis followed the PRISMA Statement (Preferred Reporting Items for Systematic Reviews and Meta-Analyses Supplementary Table S1, Supplementary Table S3). The ethical approval is not necessary for the meta-analysis of the published studies [34].

### Literature search

Systematic electronic searches were conducted in PubMed, Web of Science, Embase, CNKI (China National Knowledge Infrastructure), and Wanfang from the inception to January 15, 2016. We performed online searches using the possible Medical Subject Heading (MeSH) terms and keywords. The following search terms were used: (‘microRNA-378′ OR ‘miRNA-378′ OR ‘miR-378′ OR ‘has-mir-378′) AND (‘cancer’ OR ‘tumor’ OR ‘carcinoma’ OR ‘neoplasms’) AND (‘diagnostic’ OR ‘diagnoses’ OR ‘ROC curve’ OR ‘Diagnostic value’ OR ‘sensitivity’ OR ‘specificity’ OR receiver operating characteristics). We also retrieved the reference lists of relevant articles and reviews to identify the potentially eligible studies. Our research was restricted to Chinese and English.

### Selection criteria

Two researchers (ZZL and YYL) independently conducted the initial search, removed the duplicate records, screened the titles and abstracts forrecords, and identified records by scanning the full texts of publications. Any disagreements were resolved by fully discussion to consensus. Studies meeting the following criteria were included: (1) evaluated the diagnostic value of miR-378 for cancers; (2) Types of cancer in the studies were confirmed by gold standard. (3) Study could supply sufficient data for calculating four values (TP: true positives, FP: false positives, FN: false negatives, and TN: true negatives). Studies focused on other microRNAs, can't provide enough data were excluded. The latest studies were included for publications with duplicate data.

### Data extraction

We used a standard sheet to collect relevant data. Data extraction was conducted by ZZL and checked independently by other two authors (LFS and PC). The following data were extracted: the first author, year of publication, country, ethnicity, sample size, type of cancer, source of sample, methods of detection, values of diagnostic 4-fold contingency table (TP, FP, FN, TN). We also tried to contact the authors of articles for the missing data, and resolved the discrepancies by discussing with other authors.

### Assessment of quality

We used the Quality Assessment of Diagnostic Accuracy Studies 2(QUADAS-2) to assess the quality of included studies [35]. The QUADAS-2 tool consists of 4 key domains that discuss patient selection, index test, reference standard, and flow of patients through the study and timing of the index tests and reference standard (flow and timing). Each key domain includes two sections: risk of bias and applicability. If answers to all signaling questions for a domain are ‘yes’, then we could judge the risk of bias is low. If any question is answered ‘no’, potential bias exists. Concerns about applicability are judged as ‘low’, ‘high’, or ‘unclear’.

### Statistical analysis

First, we tested the threshold effect (Heterogeneity caused by adopting different diagnostic cutoff values when we conduct a meta-analysis of diagnostic test.) by calculating the Spearman correlation coefficient between sensitivity and specificity, If the threshold effect exists, we will combine the study results by fitting an ROC (Receiver Operating Characteristic) curve rather than pooling sensitivities and specificities or other index [36]. There is no threshold effect for the present study. We used the bivariate mixed effects models to estimate the combined sensitivity, specificity, PLRs (positive likelihood ratios), NLR (negative likelihood ratios), DORs (diagnostic odds ratios) and their 95% CI (confidence intervals) [37]. We used Q test and *I^2^* to examine the heterogeneity qualitatively and quantitatively, respectively, and *I^2^* > 50% presented the existence of heterogeneity [38]. We conducted subgroup analyses in the ethnicity (Asian vs Caucasian), cancer type (renal cell carcinoma vs other types), sample types (serum-based, plasma-based and) setting. We also calculated the area under the summary receiver operator characteristic (SROC) curve (AUC) with 95% CI. An AUC of 1.0 was judged as perfect diagnostic ability, and AUC ≤ 0.5 presents a poor diagnosis [39]. Fagan plots shows the relationship between the prior probability, the likelihood ration, and posterior test probability, and Deek's funnel plot was used to evaluate the publication bias [40]. *P* < 0.05 indicated statistically significant. All statistical analyses were performed on the Stata 12.0 station (Corp, College Station TX, USA) and Review Manager 5.3 (The Nordic Cochrane Centre, The Cochrane Collaboration, 2014.)

## SUPPLEMENTARY MATERIALS





## Abbreviations

AFP: α-fetoprotein
CEA: carcinoembryonic antigen
CA: carbohydrate antigen
PRISM: Preferred Reporting Items for Systematic Reviews and Meta-Analyses
CNKI: China National Knowledge Infrastructure
TP: true positives
FP: false positives
FN: false negatives
TN: true negatives
ROC: Receiver Operating Characteristic
SROC: summary receiver operator characteristic PLRs positive likelihood ratios
NLRs: negative likelihood ratios
DORs: diagnostic odds ratios
CI: confidence intervals
RCC: renal cell cancer
GC: gastric cancer
NC: nasopharyngeal carcinoma
CC: colorectal carcinoma
BC: breast cancer
PC: pancreatic cancer. qRT-PCR quantificational real-time polymerase chain reaction.
