# Supplementary material for: Clinical utility of microRNA-378 as early diagnostic biomarker of human cancers: a meta-analysis of diagnostic test
Source: Oncotarget. 2016 Jul 19;7(36):58569–78. doi: 10.18632/oncotarget.10707 (PMC5295453; doi:10.18632/oncotarget.10707)
Supplement: Supplementary file 1 [file oncotarget-07-58569-s001.pdf]

Clinical utility of microRNA-378 as early diagnostic biomarker of human cancers: a meta-analysis of diagnostic test

Supplementary Materials

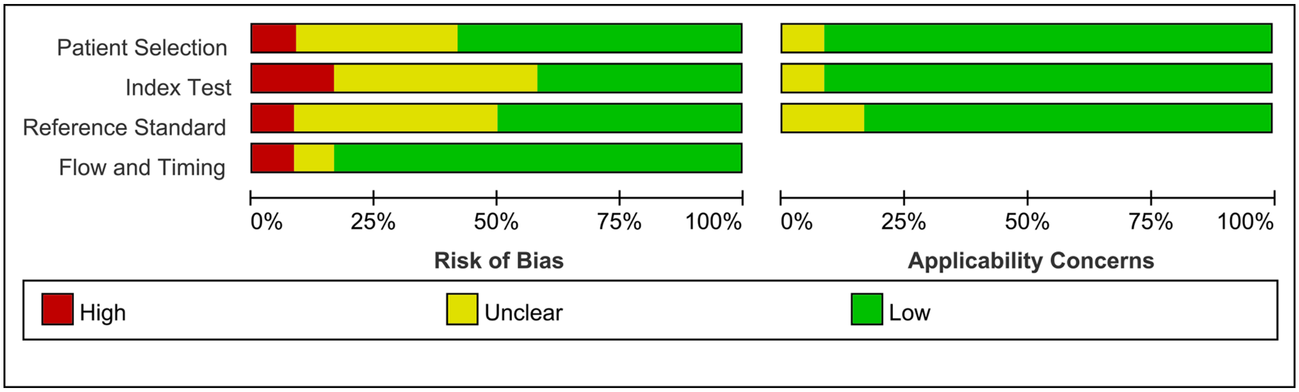

Supplementary Figure S1: Risk of bias and applicability concerns graph: review authors' judgements about each domain presented as percentages across included studies.

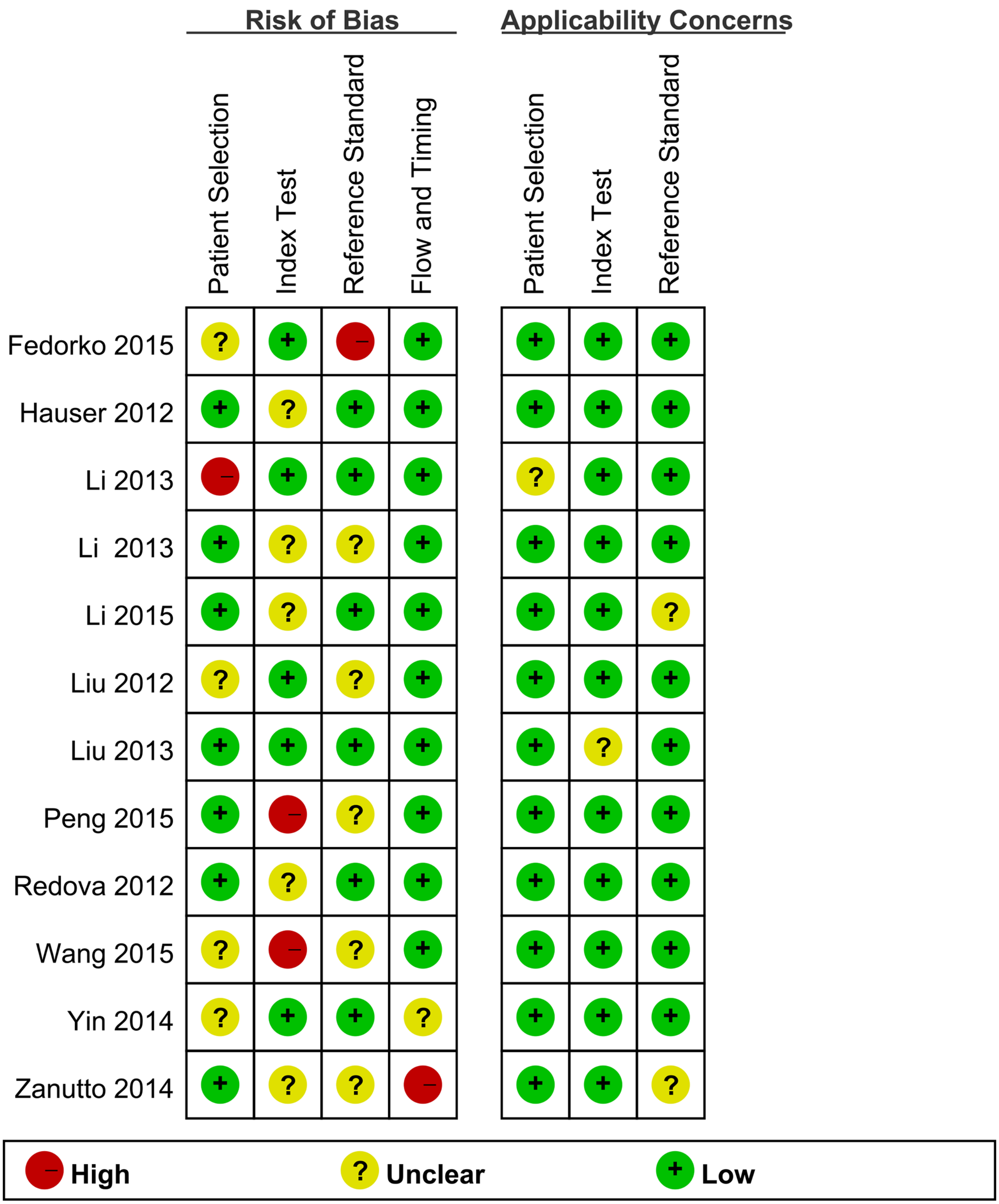

Supplementary Figure S2: Risk of bias and applicability concerns summary: review authors' judgements about each domain for each included study.

Supplementary Table S1: Preferred reporting items for systematic reviews and meta-analyses.
See Supplementary_Table_S1
